# Cost shifting or cost cutting by hospitals as a response to reimbursement reform? The case of diagnosis-related groups (DRG) scheme in China

**DOI:** 10.3389/fpubh.2025.1582001

**Published:** 2025-08-20

**Authors:** Jiabi Wang, Jinyun Zhu, Kuan Hu, Yijun Chen, Jinghua Zhang, Xiaoyu Wu

**Affiliations:** ^1^School of Accounting, Nanfang College, Guangzhou, Guangdong, China; ^2^School of Business, Macau University of Science and Technology, Macao, Macao SAR, China; ^3^Ganzhou Municipal Health Commission in Jiangxi Province of China, Ganzhou, Jiangxi, China

**Keywords:** diagnosis-related groups, cost-shifting, hospitals reimbursement reform, cataract intraocular lens (IOL) implantation, difference-in-difference

## Abstract

**Background:**

Diagnosis-related group (DRG) systems for healthcare reimbursement were recently introduced among hospitals in China, raising concerns about cost-shifting, where hospitals may increase charges for self-financing patients to offset reimbursement cuts by DRG. In 2018, both Nanchang and Ganzhou Cities in Jiangxi Province installed DRG information systems, but only Nanchang fully implemented the DRG system during the 2019–2020 pilot period.

**Materials and methods:**

Drawing from a healthcare administrative dataset of 14,310 patients' records, this study investigates the hospitalization costs associated with Intraocular Lens (IOL) implantation procedures in Jiangxi Province, China, from 2017 to 2020. By applying the quantile difference-in-differences (DID) and difference-in-difference-in-difference (DDD) methodologies, the research examines the impacts of DRG implementation on hospitalization costs, with a particular focus on self-financing patients.

**Results:**

Upon the implementation of DRG in hospitals in Nanchang, the IOL cost ratio for patients in Nanchang was lower on average by 0.047 (*p* < *0.001*), with the largest reduction observed at higher quantiles (e.g., Q75: −0.105, Q90: −0.091) (*p* < *0.001*). The DDD model revealed a decrease of 0.226 in the IOL cost ratio for self-financing patients in DRG hospitals post-implementation. These results remained robust across various healthcare cost ratios.

**Conclusion:**

The study indicates that implementing the DRG system in Chinese hospitals effectively constrained healthcare costs without causing unintended cost-shifting among self-financing patients. The system's heightened impacts on higher quantiles indicate its efficacy in addressing high-cost outliers. These outcomes are likely attributed to the centralized medical procurement and healthcare information technology integrated into the Chinese healthcare system.

## 1 Introduction

The rising financial burden of medical costs globally has prompted policymakers and the public to seek cost containment strategies (1). The prospective payment system (PPS) with diagnosis-related groups (DRG) has been widely adopted as an effective tool for controlling healthcare costs (2–8). Under this system, hospitals receive fixed payments based on patients' diagnostic classifications, eliminating the financial incentive for overtreatment (9–11). However, hospitals may engage in cost shifting, raising costs to privately insured patients to offset financial losses from reduced DRG reimbursements (12–17). The practice of cost shifting thus affects the financial wellbeing of patient groups (18–22). Empirical studies based on financial data from U.S. hospitals have found mixed results (23–26). Some studies argue against the prevalence of dynamic cost shifting (23, 27–30), while others indicate that hospitals may engage in cost shifting to mitigate financial pressures (26, 31–39).

From 2019 to 2021, China launched a national pilot project of a standardized DRG payment system among public hospitals in 30 selected cities. As the Chinese government has ordered the adoption of the DRG payment system nationwide, there has been a heated debate about the potential impacts on the financial status of hospitals and treatment outcomes of patients (11, 40–42), especially concerns regarding cost shifting to self-financing patients in China (37, 39, 43–48). However, few empirical studies have been performed testing cost shifting among Chinese hospitals, and mixed findings were reported (42, 49–52).

This study leverages a quasi-natural experiment arising from the implementation of the DRG system in Jiangxi Province, China. In 2018, both Nanchang City and Ganzhou City installed national-standard DRG information systems. However, during the pilot-experiment period from 2019 to 2021, only Nanchang City was designated to fully implement the DRG system for over 94% of disease groups in the city's public hospital system. This unique context provides a valuable research opportunity to examine the impact of DRG adoption.

Cataract intraocular lens (IOL) implantation is well-suited to studying hospital cost-shifting due to the financial incentives associated with it. Despite being a standardized procedure, significant price variation exists among IOL types (53), with patients increasingly opting for self-financed lenses for improved outcomes. These features make IOL implantation procedures a valuable context for examining cost-shifting practices.

As a traditional inland agricultural region in central-eastern China, Jiangxi Province has a high incidence of cataracts due to its lower living standard and less developed economy during the past decades. In 2009, it had ~250,000 cataract patients with 18,000 new cases annually, and blindness cases due to cataracts accounted for 0.58% of the population in this province (54). Thus, the administrative data on IOL procedure hospitalization costs from Jiangxi Province represents a valuable dataset for studying this prevalent disease in China.

By applying the difference-in-difference model to leverage the quasi-experiment of the DRG system in Jiangxi Province, this study aims to specifically examine the impact of the DRG system on the IOL cost ratio for implantation procedures, as well as the cost differences among self-financing patients.

## 2 Data and methodology

### 2.1 Data source and sampling

Data for this study were obtained from the DRG database administered by the National Healthcare Security Administration in Jiangxi Province. The selection criteria were as follows:

1) Procedure type: Cataract intraocular lens (IOL) procedures are categorized under Lens Surgery (CHS-DRG code: CS10A) and Ambulatory Lens Surgery (CHS-DRG code: CS10B).
2) Age range: this study focuses on patients aged 50 and 80. This age group is selected due to its highest prevalence of cataracts in China (55), where the incidence of cataracts among individuals over 60 years old reaches 80%, accounting for over 50% of visually disabled individuals (56). Most studies on cataract surgery ehave established age 50 as the baseline to ensure inclusivity. Patients older than 80 are excluded from this study, as they often present with more complex health conditions and experience significantly higher healthcare utilization (57).
3) Length of stay (LOS): limiting to hospital stays of one or 2 days to control for case mix and severity. Ambulatory lens surgery (CHS-DRG code: CS10B) indicates cases with 1-day hospitalization.
4) Insurance coverage: including patients covered under urban employees/resident health insurance or those self-financed, ensuring a clear and consistent sample.
5) Hospital accreditation: this study focused exclusively on cases from tertiary public hospitals with Class A accreditation, the highest accreditation level in China. Class A hospitals are recognized for their superior professional services, advanced equipment, and exceptional management skills (58). They are highly trusted by patients, regardless of the severity of their conditions (59), and are the preferred choice for most intraocular lens (IOL) procedures in the country (60). Thus, consistent and high-quality healthcare cost data can be obtained from these hospitals, ensuring reliability and comparability.
6) IOL procedure record: this study focuses exclusively on hospitalization records for patients who underwent the intraocular lens (IOL) procedure, identified by associated IOL costs and surgery charges. Follow-up visits were excluded from this analysis. As per standard practice, the IOL procedure is performed on one eye at a time. Therefore, for patients who received the IOL procedure for both eyes, there are two separate hospitalization records.
7) Procedure costs: total costs of each episode include IOL cost, surgical fees, medication expenses, operating room utilization, surgical materials, diagnostic testing, nursing services, medical equipment, and anesthesia services (61). Records exhibiting implausible cost components were excluded. Specifically, those with IOL costs falling below CNY 200 were excluded, as these may indicate inaccurate documentation or incomplete cost recording. Additionally, cases with total episode costs exceeding CNY 25,000 were excluded, since these abnormally high amounts likely resulted from complications or additional treatments beyond standard cataract surgery.
8) Study period: July 2017 to December 2020, covering 2 years before and after the experimental period.

The final dataset comprises 14,310 patients' records, encompassing demographics, clinical diagnosis, resource utilization metrics, cost data, DRG grouping factors, and policy-linked variables. All healthcare cost data were standardized to 2020 price levels using the Chinese Medical Inflation Index and are reported in Chinese Yuan (CNY) based on the original billing records. Approximate United States Dollar (USD) equivalents, provided for international reference, were calculated using the 2020 annual average exchange rate (1 USD = 6.9 CNY).

### 2.2 Statistical method

This study adopted the difference-in-difference (DID) model to evaluate the impact of the DRG payment system on the total cost of IOL implantation procedures. In 2018, both Nanchang City and Ganzhou City installed the national-standard DRG information systems and conducted comprehensive training. Meanwhile, during the experiment stage from January 2019 to January 2020, only Nanchang actually adopted the DRG system for the healthcare cost management of most diseases. Recognizing that DRG payment reforms exhibit time-lagged implementation effects, where initial policy impacts may be obscured by systemic adaptation periods. Thus, we excluded the data from August to December 2018 to avoid capturing transitional anomalies. The pre-implementation baseline was consequently defined as July 2017 to July 2018, ensuring uncontaminated measurement of pre-reform medical costs prior to DRG rollout. As a result, patients' records in Nanchang City constituted the treatment group during the post-2019 DRG implementation period. By introducing an interaction term between the post-DRG period and the treatment group indicator, we captured the DID effect.

To gain a more detailed understanding of how the DRG implementation affects medical costs across different points of the cost distribution, we further utilized the quantile difference-in-difference (QDID) model. Quantile regression allows us to estimate the treatment effect at various quantiles, especially 10th, 25th, 50th, 75th, and 90th percentiles of the cost distribution, rather than focusing solely on the mean. This approach provides several advantages. First, the quantile regression method is robust to outliers in the outcome variable, which is very common in healthcare costs. Secondly, it offers a complete view of the relationship between variables by capturing the effects at different points in the outcome distribution (62, 63). This approach allowed for better identification of heterogeneous treatment effects, offering a more nuanced understanding than focusing solely on the mean (64).

To further examine how the treatment costs of self-financing patients varied upon the implementation of the DRG policy, we adopted the Difference-in-Difference-in-Differences (DDD) method. The DDD approach extends the DID framework by incorporating an additional layer of comparison, enabling us to isolate the effect of the DRG policy on self-financing patients specifically, who are particularly susceptible to cost shifts. This method involves a triple interaction term between the post-DRG implementation period, the treatment group indicator, and a binary variable indicating self-financing status. By doing so, we can disentangle the unique impact of the DRG policy on self-financing patients from other potential confounding effects. The DDD model was similarly adjusted for patient-level and hospital-level covariates to ensure robustness. This model specifically isolates the policy impact on self-financing patients, who are particularly susceptible to cost shifts.

### 2.3 Empirical models

#### 2.3.1 DID model

The basic empirical equation has the specification as follows:


(1) $$\begin{array}{l} Y_{i,h,t}=\beta_{0}+\beta_{1}Intervention_{h}\times Post_{t}+\beta_{2}Post_{t}+\beta_{3}Intervention_{h}\\ \;+\beta_{4}Self\_financing_{i,t}+\delta X_{i,h,t}+\varepsilon_{i,h,t} \end{array}$$

where *i* represents a patient, *h* for a hospital, and *t* for a year, ε_*i, h, t*_ is a random error term.

*Y*_*i, h, t*_ represents the outcome variables: IOL cost ratio, log of IOL costs, and log of total episode costs, respectively. The IOL cost ratio indicates the proportion of the total episode costs attributed to the IOL costs. The relative ratio of intraocular lens (IOL) cost standardizes costs across diverse patient scenarios, allowing for meaningful comparisons. This approach mitigates the influence of outliers, offers a clearer understanding of typical cost distributions, and strengthens the robustness of our economic evaluations. The logarithm of IOL costs and total episode costs are adopted, because the distribution of healthcare costs is typically skewed.

The binary variable *Intervention*_*h*_ indicates an IOL case in the treatment group of Nanchang City (*Intervention*_*i, t*_ = 1), with Ganzhou (*Intervention*_*i, t*_ = 0) as the reference group. *Post*_*t*_ indicates observations from the period after DRG implementation (*Post*_*i, t*_ = 1: January 2019 to December 2020), with the period of July 2017 to July 2018 (*Post*_*i, t*_ = 0) as reference. Accordingly, the interaction term *Intervention*_*h*_×*Post*_*t*_ of DID model captures the effect of the DRG payment reform.

For a better comparison, this study placed *Self*_*financing*_*i, t*_ as a separate control variable in the equation. *Self*_*financing*_*i, t*_ is a dummy variable that *Self*_ *financing*_*i, t*_ = 0 represents patients covered by Urban Employee Basic Medical Insurance (UEBMI) and Urban Residents Basic Medical Insurance (URBMI), while *Self*_*financing*_*i, t*_ = 1 identifies patients without social health insurance.

*X*_*i, h, t*_ represents a sector that includes various patient characteristics, such as age, gender, and length of stay (LOS).

#### 2.3.2 DDD model

The DDD model is established as Equation 2.


(2) $$\begin{array}{l} Y_{i,h,t}=\beta_{0}+\beta_{1}Intervention_{h}\times Post_{t}\times Self\_financing_{i,t}\\ \;+\beta_{2}Post_{t}\times Self\_financing_{i,t}+\beta_{3}Intervention_{h}\\ \;\times Self\_financing_{i,t}+\beta_{4}Intervention_{h}\times Post_{t}+\beta_{5}Post_{t}\\ \;+\beta_{6}Intervention_{h}+\beta_{7}Self\_financing_{i,t}\\ \;+\delta X_{i,h,t}+\varepsilon_{i,h,t} \end{array}$$

The coefficient of the triple interaction term, β_1_ is the DDD estimator of interest, representing the net effect of the DRG payment reform among the self-financing patients.

Standard errors of quantile regressions were calculated by bootstrapping with 1,000 repetitions. Stata version 15.0 (Stata Corp LP, College Station, TX) was adopted for performing all statistical analyses in this study.

## 3 Empirical analysis

### 3.1 Descriptive results

Table 1 presents descriptive statistics of the analytical cohort (*N* = 14,310). Geographically, 75.49% (*n* = 10,802) of patients were treated in Nanchang vs. 24.51% (*n* = 3,508) in Ganzhou. Patients exhibited a mean age of 68.7 years, concentrated in the 70–80-year cohort (48.65%), which was consistent with cataract epidemiology in aging populations. Females comprised the majority of cases (59.13%; male-to-female ratio = 1:1.45), while reimbursement sources indicated 72.07% used medical insurance vs. 27.93% self-pay arrangements.

**Table 1:** Descriptive statistics.

**Panel A. Demographic characteristics**			
**Variables**	**Full sample** ***N*** **(%)**	**Nanchang city** ***n*** **(%)**	**Ganzhou city** ***n*** **(%)**
**Obs. Num**.	14,310 (100)	10,802 (75.49)	3,508 (24.51)
**Age (years)**			
50–59	2,165 (15.13)	1,783 (16.51)	382 (10.89)
60–69	5,155 (36.02)	3,916 (36.25)	1,239 (35.32)
70–80	6,990 (48.65)	5,103 (47.24)	1,887 (53.79)
**Gender**			
Male	5,848 (40.87)	4,252 (39.36)	1,596 (45.5)
Female	8,462 (59.13)	6,550 (60.64)	1,912 (54.5)
**Insurance status**			
Self-financing	3,998 (27.93)	2,611 (24.17)	1,387 (39.54)
Insured	10,312 (72.07)	8,191 (75.83)	2,121 (60.46)
**Panel B. Descriptive statistics of continuous variables**						
**Variables**	**Full sample**		**Nanchang city**		**Ganzhou city**	
	**Mean**	**S.D**.	**Mean**	**S.D**.	**Mean**	**S.D**.
IOL costs (CNY)	3,344^a^	1,998	4,065	1,688	1,122	980
Total episode costs (CNY)	8,728	3,856	9,943	3,347	4,987	2,756
IOL cost ratio	0.39	0.16	0.45	0.13	0.22	0.1
Length of Stay (LOS) (days)	2.58	2.33	2.5	2.27	2.83	2.47
Age (years)	68.31	7.76	67.95	7.85	69.41	7.39

^**a**^The exchange rate: 1 USD = 6.9 CNY, CNY 3344 ≈ USD 484.6; CNY 8728 ≈ USD 1264.9; CNY 4065 ≈ USD 589.1; CNY 9943 ≈ USD 1141; CNY 1122≈ USD 162.6; CNY 4987 ≈ USD 722.8.

In panel B, the IOL costs constituted, on average, 39% of total episode costs. Mean IOL costs and total episode costs per patient were CNY 3344 (USD 484.6) and CNY 8728 (USD 1264.9), respectively. Regional analysis revealed higher mean costs in Nanchang (IOL: CNY 4065, Total: CNY 9943) compared to Ganzhou (IOL: CNY 1122, Total: CNY 4987). Patients were discharged after a mean hospital length of stay of 2.58 days. All analyzed variables exhibited mean values and standard deviations within the expected range, with no significant statistical outliers identified.

### 3.2 Parallel trend test

The “parallel trend assumption” is an important prerequisite for using the DID model to judge policy effectiveness (65–68). We plotted the time trend of the mean value of Y, which was plotted by month for the intervention and control groups in a simple and intuitive way.

**Figure 1:**
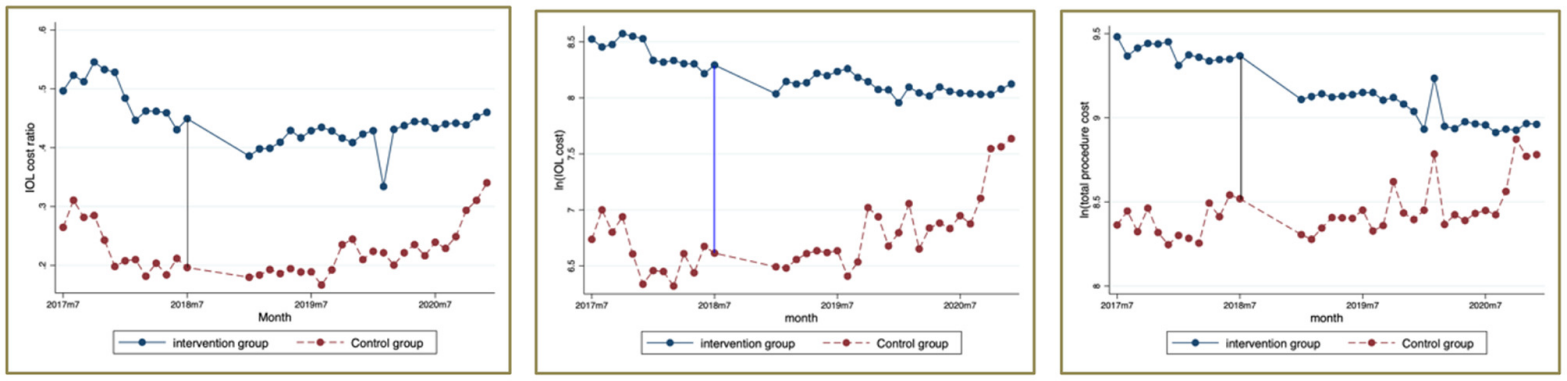
DID parallel trend test (time trend graph of the mean of Y).

Figure 1 shows the results of the DID parallel trend test. All the dependent variables between the two cities maintained a consistent trend before July 2018, while after that, the trend of the two groups showed a significant difference. That means the changes in the outcome variables between the two groups before and after the DRG's payment reform have nothing to do with the control group. Thus, the DID method can be used to evaluate the pure policy effects.

### 3.3 Regression analysis of the DID model

Table 2 reports the estimation results of the intervention's effects on three cost measures, respectively. Panel A indicates that the intervention significantly reduced the IOL cost ratio post-implementation. As reported in column 1, the estimated coefficients of the interaction term (Intervention × Post) are negative and statistically significant on average and across most quantiles. This indicates that post-DRG implementation, the average IOL cost ratio in Nanchang hospitals declined by 0.047 (4.7 percentage points) relative to Ganzhou. It shows DRG effectively contained IOL-related expenditures without increasing total hospitalization costs, demonstrating its efficacy in targeted cost control. The largest reduction is observed at higher quantiles (e.g., Q75: −0.105, Q90: −0.091), with maximal declines occurring among high-cost patients where financial reallocation was most probable. For the age group, the results in column 1 are not significant at the 60–69-year range; however, the IOL cost ratio is relatively slightly decreased at the 75th and 90th percentiles in older patients, which is significant at the 1% confidence level. The IOL cost ratio of self-financing patients slightly decreased by 3.1 percentage points after DRG reform at the 1% significance level, and the decline was even greater at higher quantiles (e.g., Q75: −0.038, Q90: −0.047).

**Table 2:** Result of DID estimates (OLS and quantile regressions)

**Panel A. Regression results on IOL cost ratio**						
**Variables**	**(1)**	**(2)**	**(3)**	**(4)**	**(5)**	**(6)**
	**OLS**	**Q10**	**Q25**	**Q50**	**Q75**	**Q90**
Intervention^*^Post	−0.047^***a^	−0.017^***^	−0.007	−0.055^***^	−0.105^***^	−0.091^***^
	(0.005)	(0.005)	(0.008)	(0.004)	(0.006)	(0.007)
Intervention	0.254^***^	0.222^***^	0.267^***^	0.324^***^	0.296^***^	0.264^***^
	(0.004)	(0.004)	(0.006)	(0.004)	(0.004)	(0.005)
Post	−0.025^***^	−0.018^***^	−0.039^***^	−0.009^***^	0.011^**^	−0.023^***^
	(0.004)	(0.004)	(0.006)	(0.004)	(0.005)	(0.008)
Self-financing	−0.031^***^	−0.006	−0.021^***^	−0.02^***^	−0.038^***^	−0.047^***^
	(0.002)	(0.004)	(0.004)	(0.003)	(0.003)	(0.004)
LOS (days)	−0.012^***^	−0.018^***^	−0.018^***^	−0.015^***^	−0.011^***^	−0.01^***^
	(0.001)	(0.001)	(0.001)	(0.001)	(0.001)	(−0.001)
**Age (years)**						
60–69	−0.004	0.005	0.002	−0.006^*^	−0.01^***^	−0.015^***^
	(0.003)	(0.004)	(0.003)	(0.003)	(0.003)	(0.004)
70–80	−0.006^*^	0.006	0.001	−0.007^**^	−0.015^***^	−0.021^***^
	(0.003)	(0.004)	(0.002)	(0.003)	(0.003)	(0.004)
**Gender**						
Male	0.005^**^	0.001	0.008^**^	0.004^*^	0.003^*^	0
	(0.002)	(0.001)	(0.003)	(0.002)	(0.001)	(0.003)
Constant	0.283^***^	0.176^***^	0.207^***^	0.224^***^	0.329^***^	0.443^***^
	(0.005)	(0.006)	(0.006)	(0.005)	(0.004)	(0.008)
R-squared	0.464	0.27	0.393	0.338	0.267	0.244
**Panel B. Regression results on ln (IOL costs)**						
**Variables** ^b^	**(1)**	**(2)**	**(3)**	**(4)**	**(5)**	**(6)**
	**OLS**	**Q10**	**Q25**	**Q50**	**Q75**	**Q90**
Intervention^*^Post	−0.425^***^	−0.255^***^	−0.209^***^	−0.527^***^	−0.943^***^	−0.286^***^
	(0.029)	(0.017)	(0.019)	(0.009)	(0.022)	(0.029)
Intervention	1.767^***^	1.870^***^	2.231^***^	2.299^***^	1.829^***^	1.136^***^
	(0.021)	(0.014)	(0.011)	(0.007)	(0.015)	(0.023)
Post	0.163^***^	−0.023^*^	−0.035^***^	0.272^***^	0.704^***^	−0.032
	(0.027)	(0.013)	(0.016)	(0.008)	(0.02)	(0.029)
Self-financing	−0.130^***^	0.001	−0.015	−0.019^***^	−0.197^***^	−0.064^***^
	(0.012)	(0.013)	(0.01)	(0.006)	(0.018)	(0.012)
Constant	6.567^***^	6.007^***^	6.055^***^	6.049^***^	6.838^***^	7.736^***^
	(0.027)	(0.025)	(0.014)	(0.01)	(0.018)	(0.029)
R-squared	0.587	0.45	0.545	0.386	0.258	0.234
**Panel C. Regression results on ln (total episode cost)**						
**Variables** ^b^	**(1)**	**(2)**	**(3)**	**(4)**	**(5)**	**(6)**
	**OLS**	**Q10**	**Q25**	**Q50**	**Q75**	**Q90**
Intervention^*^Post	−0.344^***^	−0.318^***^	−0.298^***^	−0.221^***^	−0.713^***^	−0.319^***^
	(0.013)	(0.01)	(0.006)	(0.045)	(0.014)	(0.019)
Intervention	0.983^***^	1.028^***^	1.096^***^	1.078^***^	1.151^***^	0.786^***^
	(0.009)	(0.008)	(0.005)	(0.007)	(0.01)	(0.018)
Post	0.061^***^	−0.006	−0.032^***^	−0.067^*^	0.440^***^	0.077^***^
	(0.012)	(0.006)	(0.004)	(0.044)	(0.013)	(0.017)
Self–financing	−0.058^***^	−0.001	−0.009^***^	−0.024^***^	−0.076^***^	−0.075^***^
	(0.005)	(0.006)	(0.004)	(0.008)	(0.009)	(0.01)
Constant	7.952^***^	8.013^***^	8.118^***^	8.216^***^	8.687^***^	7.952^***^
	(0.001)	(0.008)	(0.006)	(0.013)	(0.015)	(0.021)
*R*–squared	0.712	0.61	0.587	0.461	0.383	0.38

^**a**^Standard errors, clustered by hospital, are in parentheses. ^*^P < 0.05, ^**^P < 0.01, ^***^P < 0.001.

^**b**^Although not detailed in the tables, the regression specifications in Panels B and C are consistent with the variables included in Panel A. The complete results are provided in Appendix Tables A.1, A.2.

**Figure 2:**
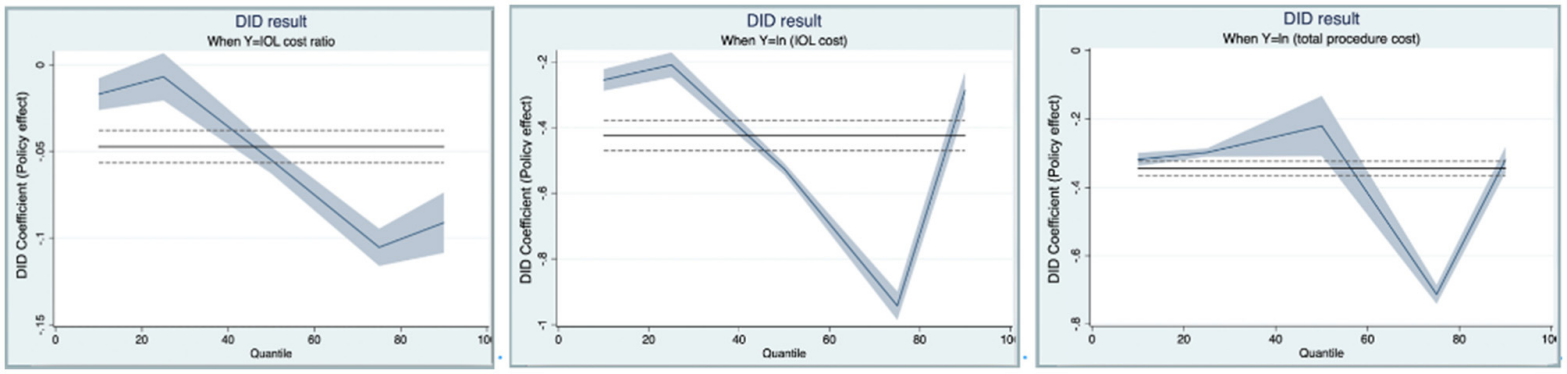
Quantile regression results of the DID model.

The findings reported in Panels B and C keep the key results. The results show that IOL costs and total episode costs have significantly decreased in the intervention group (Nanchang city) after DRG reform by ~4.25 and 3.44 percentage points at the 1% significance level, respectively. Notably, these two costs fell the most at the 75th percentile. The full results were shown in Appendix Tables A.1, A.2. These effects are visualized in Figure 2.

### 3.4 Regression analysis of the DDD model

As reported in Table 3, for the IOL cost ratio, the coefficient of the triple interaction term is −0.226 (*p* < 0.001), indicating a reduction in the cost ratio for self-financing patients in the treatment group post-policy period. Across all three dependent variables, the coefficient for the triple interaction term is negative and highly significant at the 1% level. While the IOL costs and total episode costs of self-financing patients were also significantly decreased, more than those of insured patients, after DRG's payment reform in Nanchang city. This resulted in the DRG policy curbing the growth of medical costs for self-financing patients.

**Table 3:** Key results of DDD estimates (OLS).

**Variables^a^**	**(1)**	**(2)**	**(3)**
	**IOL cost ratio**	**ln (IOL costs)**	**ln (total episode costs)**
Intervention^*^Post ^*^Self-financing	−0.226^***^	−1.676^***^	−0.853^***^
	(0.011)^b^	(0.067)	(0.03)
Intervention^*^Post	0.1^***^	0.608^***^	0.160^***^
	(0.007)	(0.04)	(0.019)
Intervention^*^ Self-financing	0.189^***^	1.346^***^	0.674^***^
	(0.008)	(0.04)	(0.018)
Post^*^ Self-financing	0.248^***^	1.702^***^	0.805^***^
	(0.01)	(0.063)	(0.029)
Intervention	0.106^***^	0.747^***^	0.488^***^
	(0.006)	(0.035)	(0.016)
Post	−0.179^***^	−0.881^***^	−0.433^***^
	(0.006)	(0.039)	(0.018)
Self-financing	−0.217^***^	−1.383^***^	−0.651^***^
	(0.006)	(0.036)	(0.016)
Constant	0.435^***^	7.594^***^	8.716^***^
	(0.007)	(0.039)	(0.017)
Observations	14,310	14,310	14,310
R-squared	0.491	0.625	0.742

^a^Although not detailed, the regression specifications in this table are consistent with the variables included in Table 2. The results of the full model are in Appendix Table A.3.

^b^Standard errors, clustered by hospital, are in parentheses. ^***^P < 0.001.

**Figure 3:**
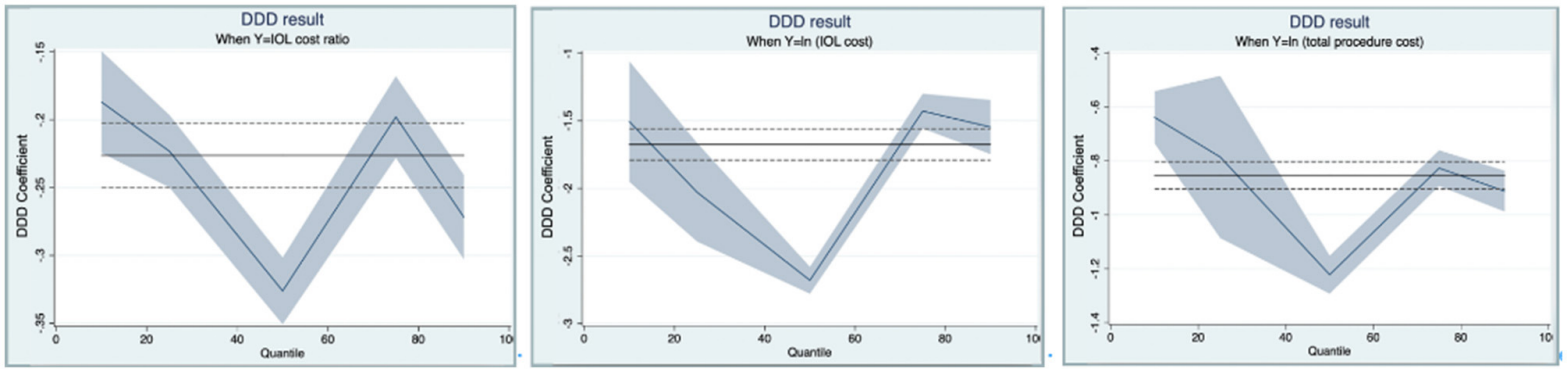
Quantile regression results of the DDD model on triple interaction terms.

Figure 3 shows the quantile regression results of the DDD model on triple interaction terms. As illustrated in Figure 3, the policy effects among self-financing patients varied across quantiles, with the largest reduction at the 50th percentile (refer to Appendix Table A.4).

### 3.5 Robustness check

To assess whether significant differences exist across the quantiles, we conducted interquartile tests by comparing other percentiles with the 50th percentile using the *iqreg* command in STATA (Appendix Table A.5).

An additional test incorporating observations from August to December 2018, which corresponds to the introduction period of DRG, was excluded from the main analysis. The results remained consistent (Appendix Table A.6).

## 4 Discussion

### 4.1 The cost-control effect of DRG policy

Applying the DID models, this study consistently found that the implementation of the DRG system in Nanchang City significantly reduced healthcare costs as measured by the ratio of IOL in the total cost of hospitalization. The results of this study align with prior research (11, 48, 49, 69–73). This might be because the price for a particular disease, including healthcare services, drugs, and medical consumables, was predetermined after DRG. Thus, doctors will concentrate more on the principal diagnosis and principal treatment procedure (11). Unlike the previous fee-for-service system, the case-based policy could motivate doctors to standardize diagnosis and treatment, and physician-induced overtreatment and medical costs could also be reduced.

Furthermore, the quantile regression results revealed substantial heterogeneity in the intervention's impact, with stronger effects observed at higher quantiles, where overtreatment and unnecessary resource utilization are most likely to occur (11). It might be because doctors are more likely to avoid unnecessary high-value medical treatment than induce patients to use higher-priced lenses after DRG reform. A previous study showed that healthcare providers would reduce overtreatment behavior after DRGs' reform (11). These findings underscore the dual value of the DRG system, a perspective that has not yet been addressed in the existing literature.

### 4.2 Impact of DRG reform on insured and self-financing patients and cost-shifting dynamics

The findings in the DDD model suggested that after the implementation of the DRG system in Nanchang City, the IOL cost ratio of self-financing patients was significantly lower by about twenty-three percentage points when compared with those having social healthcare insurance, ceteris paribus. This could be considered evidence that the cost-control effect of the DRG policy was more focused on self-financing patients and that the effect of the DRG policy was more pronounced among patients without social security. Thus, this empirical evidence did not support the cost-shifting hypothesis.

This result was consistent with studies in China (49, 52). However, some studies in China found opposite results: cost shifting to self-financing patients, uninsured, older, or more complicated patients after reform of the medical system (37, 39, 44, 46–48, 74). This finding also contrasted with previous literature in developed countries discussing cost-shifting, where hospitals shift part of medical costs to self-financing patients to cover losses from government cutbacks (15, 35, 36).

The cost-shifting could happen in developed countries, probably because it is popular for self-financing patients to purchase commercial insurance, which is likely the target of healthcare cost-shifting. The medical costs shifted to self-financing patients will ultimately be borne by insurance companies. In China, it's a different story. China's health insurance participation rate remains above 95% (75), and fewer patients purchase commercial insurance. Thus, if hospitals shift medical costs to self-financing patients, these costs will probably be borne by the patients themselves. For those self-financing patients, some patients do not have medical insurance, some patients are high-net-worth individuals who use expensive imported lenses, which are not reimbursable by medical insurance, and some patients take interprovincial medical treatment, so they can't use their medical insurance card. Self-financing patients, who bear the full burden of medical expenses out-of-pocket, tend to exhibit greater price sensitivity (76). Moreover, China's doctor-patient relationship has become more tense in recent years, and doctors may be less likely to induce high-priced medical services to avoid disputes. At the same time, after the implementation of centralized procurement of lenses, the prices of medical consumables are more open and transparent, the medical costs are under supervision of big data technology, any excessive self-financing ratios might display abnormal status in the health insurance system which will attract official investigation, thus for those self-financing patients, physicians may be less likely to induce extravagant health services. For these reasons, medical costs are not easy to pass on, and cost-shifting is also more challenging to implement.

Moreover, these positive spill-over effects of the DRG system on self-financing patients may be owing to the concurrent centralized medical procurement and healthcare information technology in China, which have improved pricing transparency for medical consumables (77, 78).

### 4.3 Strengths and limitations

This research delivers several field-advancing contributions to the health policy literature. First, by integrating QDID with the DDD method, the study detected distributional effects across patient subgroups in policy evaluation. Second, the research validated the spill-over effect on self-financing patients, disproving universal cost-shifting assumptions and establishing DRG as an equity-enabling policy. Third, the cost-saving effect verified in the research enables global policymakers, especially developing countries and low- and middle-income countries, to codify cross-group equity into further medical reform.

The study is subject to several limitations, which offer opportunities for future research. First, the primary limitation of this study lies in the data, which is derived solely from public hospitals within a province with a less developed economy in China. Findings may not generalize to wealthy provinces or private hospitals, where financial incentives and patient demographics differ. Further research should test DRG's impact across diverse regions and hospital types to assess generalizability. Due to data limitations, this study was unable to account for patient comorbidities or socioeconomic status. However, this is expected to introduce minimal bias, as the sample was restricted to a homogeneous, low-risk group undergoing standardized cataract surgery. Thus, the cost changes identified are attributable to physician behaviors and policy impacts. Future studies may explore variabilities further when comorbidity and socioeconomic data become available. Second, the study only focused on IOL implantation, a high-volume but technically standardized procedure. The findings of this study may not apply to complex, variable-cost procedures. Future research should broaden its scope to high-cost, heterogeneous procedures to identify whether DRG's anti-cost-shifting effect holds universally. Finally, reimbursement cuts could theoretically impact care quality despite efforts to constrain costs. This study did not assess the long-term impacts on clinical outcomes or patient satisfaction. Future research should track longitudinal data to evaluate comprehensive health outcomes.

## 5 Conclusion

This study investigates the impact of diagnosis-related groups (DRG) reimbursement systems on hospitalization costs in China, using the cases of Intraocular Lens (IOL) implantation procedures across two major cities in Jiangxi Province of China from 2017 to 2020.

DRG significantly reduced the IOL cost ratio. Crucially, maximal reductions occurred precisely where cost-shifting risk was highest, disproving concerns that hospitals would inflate charges for complex or high-cost cases. While the DDD estimator revealed a reduction in self-financing patients' IOL cost ratio post-DRG, reflected DRG policy brought a spill-over effect to them, which means the unintended yet beneficial consequence wherein DRG's cost-containment mechanisms disproportionately protected self-financing patients instead of triggering financial burden transfers. DRG policy also compressed hospitalization costs and established DRG reimbursement as a cost containment instrument for the medical system. These empirical findings suggest that DRG policies, when coupled with strong regulatory oversight and information technology support, can achieve their cost-containment goals effectively without major cost-shifting issues.

Given these positive outcomes, the study supports ongoing policy advancements to strengthen DRG mechanisms further and maintain high levels of governance transparency in the healthcare industry. Future research should expand these findings across varied clinical and regional contexts.

## Data Availability

The data analyzed in this study is subject to the following licenses/restrictions: Relevant administrative permissions were received from the Ganzhou Municipal Provincial Health Committee of China, who provided dataset for the study. Any prospective user of the original dataset should apply for separate permission directly from the health administration authority. Requests to access these datasets should be directed to Jinyun Zhu, gzswjwjgdw@ganzhou.gov.cn.
