# Supplementary material for: Cost shifting or cost cutting by hospitals as a response to reimbursement reform? The case of diagnosis-related groups (DRG) scheme in China
Source: Front Public Health. 2025 Aug 20;13:1582001. doi: 10.3389/fpubh.2025.1582001 (PMC12404921; doi:10.3389/fpubh.2025.1582001)
Supplement: Supplementary file 1 [file Table_1.docx]

Supplementary Material

# Supplementary Tables

**Appendix Table A.1**

**Full result of OLS and Quantile DID estimates when Y= ln (IOL costs)**

| VARIABLES | (1) | (2) | (3) | (4) | (5) | (6) |
| --- | --- | --- | --- | --- | --- | --- |
|  | OLS | Q10 | Q25 | Q50 | Q75 | Q90 |
| Intervention*Post | -0.425*** | -0.255*** | -0.209*** | -0.527*** | -0.943*** | -0.286*** |
|  | (0.029)^1^ | (0.017) | (0.019) | (0.009) | (0.022) | (0.029) |
| Intervention | 1.767*** | 1.87*** | 2.231*** | 2.299*** | 1.829*** | 1.136*** |
|  | (0.021) | (0.014) | (0.011) | (0.007) | (0.015) | (0.023) |
| Post | 0.163*** | -0.023* | -0.035*** | 0.272*** | 0.704*** | -0.032 |
|  | (0.027) | (0.013) | (0.016) | (0.008) | (0.02) | (0.029) |
| Self-financing | -0.130*** | 0.001 | -0.015 | -0.019*** | -0.197*** | -0.064*** |
|  | (0.012) | (0.013) | (0.01) | (0.006) | (0.018) | (0.012) |
| LOS (days) | 0.044*** | 0.008* | 0 | 0.033*** | 0.037*** | 0.04*** |
|  | (0.002) | (0.004) | (0.002) | (0.002) | (0.004) | (0.003) |
| **Age (years)** |  |  |  |  |  |  |
| 50-59 | 1 | 1 | 1 | 1 | 1 | 1 |
| 60-69 | -0.033** | 0.012 | -0.004 | -0.013 | -0.065*** | -0.061*** |
|  | (0.016) | (0.015) | (0.01) | (0.007) | (0.024) | (0.019) |
| 70-80 | -0.056*** | 0.012 | -0.004 | -0.019** | -0.111*** | -0.065*** |
|  | (0.015) | (0.015) | (0.01) | (0.008) | (0.023) | (0.019) |
| **Gender** |  |  |  |  |  |  |
| Female | 1 | 1 | 1 | 1 | 1 | 1 |
| Male | 0.013 | -0.000* | 0 | 0.006 | 0.001 | 0 |
|  | (0.01) | (0) | (0.003) | (0.004) | (0.01) | (0.004) |
| Constant | 6.567*** | 6.007*** | 6.055*** | 6.049*** | 6.838*** | 7.736*** |
|  | (0.027) | (0.025) | (0.014) | (0.01) | (0.018) | (0.029) |
| R-squared | 0.587 | 0.45 | 0.545 | 0.386 | 0.258 | 0.234 |

***Note1:*** *Standard errors, clustered by hospital, are in parentheses. *P < 0.05, **P < 0.01, ***P < 0.001.*

**Appendix Table A.2**

**Full result of OLS and Quantile DID estimates when Y= ln (total episode costs)**

| VARIABLES | (1) | (2) | (3) | (4) | (5) | (6) |
| --- | --- | --- | --- | --- | --- | --- |
|  | OLS | Q10 | Q25 | Q50 | Q75 | Q90 |
| Intervention*Post | -0.344*** | -0.318*** | -0.298*** | -0.221*** | -0.713*** | -0.319*** |
|  | (0.013)^1^ | (0.01) | (0.006) | (0.045) | (0.014) | (0.019) |
| Intervention | 0.983*** | 1.028*** | 1.096*** | 1.078*** | 1.151*** | 0.786*** |
|  | (0.009) | (0.008) | (0.005) | (0.007) | (0.01) | (0.018) |
| Post | 0.061*** | -0.006 | -0.032*** | -0.067* | 0.440*** | 0.077*** |
|  | (0.012) | (0.006) | (0.004) | (0.044) | (0.013) | (0.017) |
| Self-financing | -0.058*** | -0.001 | -0.009*** | -0.024*** | -0.076*** | -0.075*** |
|  | (0.005) | (0.006) | (0.004) | (0.008) | (0.009) | (0.01) |
| LOS (days) | 0.073*** | 0.054*** | 0.057*** | 0.07*** | 0.075*** | 0.083*** |
|  | (0.001) | (0.002) | (0.001) | (0.002) | (0.002) | (0.002) |
| **Age (years)** |  |  |  |  |  |  |
| 50-59 | 1 | 1 | 1 | 1 | 1 | 1 |
| 60-69 | -0.028*** | 0.006 | -0.011*** | -0.034*** | -0.040*** | -0.041*** |
|  | (0.007) | (0.005) | (0.005) | (0.01) | (0.012) | (0.013) |
| 70-80 | -0.059*** | 0 | -0.018*** | -0.044*** | -0.074*** | -0.079*** |
|  | (0.007) | (0.004) | (0.005) | (0.009) | (0.011) | (0.012) |
| **Gender** |  |  |  |  |  |  |
| Female | 1 | 1 | 1 | 1 | 1 | 1 |
| Male | 0.005 | 0 | 0 | 0.001 | 0.003 | 0.003 |
|  | (0.004) | (0.002) | (0.002) | (0.003) | (0.004) | (0.006) |
| Constant | 8.23*** | 8.013*** | 8.118*** | 8.216*** | 8.687*** | 7.952*** |
|  | (0.012) | (0.008) | (0.006) | (0.013) | (0.015) | (0.02) |
| R-squared | 0.712 | 0.61 | 0.587 | 0.461 | 0.383 | 0.38 |

***Note1:*** *Standard errors, clustered by hospital, are in parentheses. *P < 0.05, **P < 0.01, ***P < 0.001.*

**Appendix Table A.3**

**Full results of DDD estimates (OLS)**

| VARIABLES | (1) | (2) | (3) |
| --- | --- | --- | --- |
|  | IOL cost ratio | ln (IOL costs) | ln (total episode costs) |
| Intervention*Post*Self-financing | -0.226*** | -1.676*** | -0.853*** |
|  | (0.011) ^1^ | (0.067) | (0.03) |
| Intervention*Post | 0.1*** | 0.608*** | 0.160*** |
|  | (0.007) | (0.04) | (0.019) |
| Intervention* Self-financing | 0.189*** | 1.346*** | 0.674*** |
|  | (0.008) | (0.04) | (0.018) |
| Post* Self-financing | 0.248*** | 1.702*** | 0.805*** |
|  | (0.01) | (0.063) | (0.029) |
| Intervention | 0.106*** | 0.747*** | 0.488*** |
|  | (0.006) | (0.035) | (0.016) |
| Post | -0.179*** | -0.881*** | -0.433*** |
|  | (0.006) | (0.039) | (0.018) |
| Self-financing | -0.217*** | -1.383*** | -0.651*** |
|  | (0.006) | (0.036) | (0.016) |
| LOS (days) | -0.015*** | 0.028*** | 0.065*** |
|  | (0) | (0.002) | (0.001) |
| Age (years) |  |  |  |
| 50-59 | 1 | 1 | 1 |
| 60-69 | -0.002 | -0.021 | -0.022*** |
|  | (0.003) | (0.015) | (0.006) |
| 70-80 | -0.002 | -0.028* | -0.045*** |
|  | (0.003) | (0.015) | (0.006) |
| Gender |  |  |  |
| Female | 1 | 1 | 1 |
| Male | 0.004** | -0.01 | -0.003 |
|  | (0.002) | (0.009) | (0.004) |
| Constant | 0.435*** | 7.594*** | 8.716*** |
|  | (0.007) | (0.039) | (0.017) |
| R-squared | 0.491 | 0.625 | 0.742 |

***Note1:*** *Standard errors, clustered by hospital, are in parentheses. *P < 0.05, **P < 0.01, ***P < 0.001.*

**Appendix Table A.4**

**Quantile regression results of the DDD model on triple interaction terms^1^**

| Intervention*Post*Self-financing | | (1) | (2) | (3) | (4) | (5) |
| --- | --- | --- | --- | --- | --- | --- |
|  |  | Q10 | Q25 | Q50 | Q75 | Q90 |
| IOL cost ratio |  | -0.187*** | -0.223*** | -0.326*** | -0.02*** | -0.272*** |
|  |  | (0.019)^2^ | (0.013) | (0.013) | (0.015) | (0.016) |
| Ln (IOL costs) |  | -1.506*** | -2.028*** | -2.679*** | -1.429*** | -1.547*** |
|  |  | (0.227) | (0.186) | (0.051) | (0.066) | (0.102) |
| Ln (total episode costs) | | -0.638* | -0.785*** | -1.222*** | -0.827*** | -0.912 |
|  |  | (0.049) | (0.154) | (0.036) | (0.034) | (0.039) |

***Note1:*** *This table only show the main results of Quantile regression of the DDD model.*

***Note2:*** *Standard errors, clustered by hospital, are in parentheses. *P < 0.05, **P < 0.01, ***P < 0.001.*

**Appendix Table A.5**

**Interquartile effects**

| **Panel A** | Interquartile |  |  |  |
| --- | --- | --- | --- | --- |
| ln (IOL cost ratio) | 0.1～0.5 | 0.25～0.5 | 0.5～0.75 | 0.5～0.9 |
| Intervention*Post | -0.038*** | -0.048*** | -0.05*** | -0.036*** |
|  | (0.005)^1^ | (0.007) | (0.005) | (-0.013) |
| Intervention | 0.101*** | 0.056*** | -0.28*** | -0.06*** |
|  | (0.004) | (0.006) | (0.004) | (-0.011) |
| Post | 0.008* | 0.03*** | 0.02*** | -0.014 |
|  | (0.004) | (0.005) | (0.004) | (-0.013) |
| **Panel B** |  |  |  |  |
| ln (IOL costs) | 0.1～0.5 | 0.25～0.5 | 0.5～0.75 | 0.5～0.9 |
| Intervention*Post | -0.271*** | -0.317*** | -0.416*** | 0.24*** |
|  | (0.02) | (0.019) | (0.019) | (0.029) |
| Intervention | 0.428*** | 0.068*** | -0.47*** | -1.163*** |
|  | (0.02) | (0.013) | (0.01) | (0.02) |
| Post | 0.295*** | 0.307*** | 0.432*** | -0.304*** |
|  | (0.013) | (0.015) | (0.017) | (0.028) |
| **Panel C** |  |  |  |  |
| ln (total episode costs) | 0.1～0.5 | 0.25～0.5 | 0.5～0.75 | 0.5～0.9 |
| Intervention*Post | 0.098 | 0.077* | -0.492*** | -0.099*** |
|  | (0.043) | (0.039) | (0.026) | (0.042) |
| Intervention | 0.05*** | -0.018*** | 0.073*** | -0.291*** |
|  | (0.008) | (0.005) | (0.008) | (0.022) |
| Post | -0.06 | -0.035 | 0.507*** | 0.144*** |
|  | (0.043) | (0.039) | (0.028) | (0.04) |

***Note1:*** **P < 0.05, **P < 0.01, ***P < 0.001.*

**Appendix Table A.6**

**Robustness Test, the OLS Results of DID estimates with full data (N=16,865)** ^2^

| VARIABLES | (1) | (2) | (3) |
| --- | --- | --- | --- |
|  | IOL cost ratio | ln (IOL cost ) | ln (total procedure cost ) |
| Intervention*Post | -0.049*** | -0.386*** | -0.288*** |
|  | (0.004) ^1^ | (0.026) | (0.012) |
| Intervention | 0.257*** | 1.736*** | 0.929*** |
|  | (0.003) | (0.017) | (0.008) |
| Post | -0.014*** | 0.158*** | 0.018*** |
|  | (0.004) | (0.025) | (0.011) |
| Self-financing | -0.036*** | -0.183*** | -0.085*** |
|  | (0.002) | (0.012) | (0.005) |
| LOS (days) | -0.011*** | 0.047*** | 0.073*** |
|  | (0) | (0.002) | (0.001) |
| Age (years) |  |  |  |
| 50-59 | 1 | 1 | 1 |
| 60-69 | -0.004 | -0.034** | -0.031*** |
|  | (0.003) | (0.015) | (0.006) |
| 70-80 | -0.006** | -0.06*** | -0.064*** |
|  | (0.003) | (0.014) | (0.006) |
| Gender |  |  |  |
| Female | 1 | 1 | 1 |
| Male | 0.003* | 0.008 | 0.002 |
|  | (0.002) | (0.009) | (0.004) |
| Constant | 0.271*** | 6.573*** | 8.282*** |
|  | (0.004) | (0.024) | (0.01) |
| R-squared | 0.489 | 0.616 | 0.726 |

***Note1:*** *Standard errors, clustered by hospital, are in parentheses. *P < 0.05, **P < 0.01, ***P < 0.001.*

***Note2:*** *The robustness test using full data including sample from August to December 2018, total sample size are 16,865.*
